# Population pharmacokinetics of lenalidomide in Chinese patients with influence of genetic polymorphisms of ABCB1

**DOI:** 10.1038/s41598-024-52460-2

**Published:** 2024-01-31

**Authors:** Xiaoxiao Liang, Haiyan Shi, Kehong Bi, Saran Feng, Shixian Chen, Wei Zhao, Xin Huang

**Affiliations:** 1 Department of Clinical Pharmacy, The First Affiliated Hospital of Shandong First Medical University & Shandong Provincial Qianfoshan Hospital, Shandong Medicine and Health Key Laboratory of Clinical Pharmacy, Jinan, Shandong China; 2 School of Pharmacy, Shandong University of Traditional Chinese Medicine, Jinan, Shandong China; 3 Department of Hematology, The First Affiliated Hospital of Shandong First Medical University & Shandong Provincial Qianfoshan Hospital, Jinan, Shandong China

**Keywords:** Lymphoma, Myelodysplastic syndrome, Myeloma

## Abstract

Affected by differences in the pharmacokinetics (PK) of lenalidomide, the toxicity of lenalidomide varies among different patients, with serious toxicity leading to dose reduction or discontinuation. The differences in the PK of lenalidomide may be related to factors such as patients’ physiological characteristics, pathological characteristics and gene polymorphisms etc., which may also affect its toxicity. The aim of this study is to establish a population pharmacokinetic (PPK) model of lenalidomide and explore factors associated with the adverse events (AEs) of lenalidomide from a PK perspective. Blood samples were collected by opportunistic blood collection. Drug concentrations were determined by using HPLC/MS and genotype of ABCB1 3435 C > T (rs1045642), ABCB1 1236 A > G (rs1128503) and ABCB1 2677 A > C/T (rs2032582) was tested by the first-generation DNA sequencing technology. NONMEM software and SPSS 26.0 software were used respectively to establish PPK model of lenalidomide and explore the correlation between PK parameters and the incidence of serious AEs of lenalidomide. 51 patients were enrolled in the PPK study, and one-compartment model with first-order absorption and elimination agreed well with the observed data. The significant covariate affecting lenalidomide apparent volume of distribution (V/F) were the gene polymorphism of ABCB1 3435 C > T and diet. Safety studies could be conducted in 39 patients. The V/F value in patients suffering from serious AEs was significantly higher than that in others ( median = 67.04 L vs 37.17 L, *P* = 0.033). According to the covariates screened, the incidence of serious AEs was higher in patients with genotype CT or TT at ABCB1 3435 C > T locus than that in patients with genotype CC (*P* = 0.039). Additionally, V/F value was the highest in patients carrying genotype TT with postprandial medication, in whom the incidence of serious AEs was higher than others (*P* = 0.037). In conclusion, the genotype of ABCB1 3435 C > T locus and diet had pharmacokinetically relevant impact on lenalidomide, which may also be related to the incidence of serious AEs. Patients with gene variants of CT or TT at ABCB1 3435 C > T locus may be more susceptible to serious AEs, and monitoring of adverse reactions should be particularly strengthened in patients who carried genotype TT with postprandial medication.

## Introduction

Lenalidomide, a structural derivative of thalidomide, was initially approved in the United States in 2005 for the treatment of transfusion-dependent anemia caused by myelodysplastic syndromes (MDS) associated with 5q deletion (del 5q). A year later, lenalidomide was also approved for the treatment of multiple myeloma (MM), which has proved to show significant efficacy. Furthermore, owing to its direct or indirect anti-tumor effects against malignant lymphocytes, lenalidomide also demonstrates certain efficacy against hematologic malignancies^1^, including mantle cell lymphoma (MCL), marginal zone lymphoma (MZL), follicular lymphoma (FL), diffuse large B-cell lymphoma (DLBCL), chronic lymphocytic leukemia (CLL) and so on^2–10^. Compared to thalidomide, lenalidomide exhibits better efficacy without inducing adverse reactions such as drowsiness or teratogenicity, so it has gradually replaced thalidomide as the preferred immunomodulatory drug (IMiD)^11–13^.

As it is not a substrate, inhibitor or inducer of cytochrome P450, lenalidomide is found to be minimally metabolized by the liver and eliminated predominantly via urinary excretion with an approximate half-life (t_1/2_) of 3–5 h, though it can be prolonged threefold in patients with moderate to serious renal impairment ^14–16^. Noteworthily, lenalidomide is a weak substrate of P-glycoprotein (P-gp)^17^. In some case reports, it is observed that P-gp inhibitors significantly increase the absorption of lenalidomide in patients with MM^18,19^. Since P-gp is encoded by the gene of ATP-binding cassette subfamily B member 1 (ABCB1), the genetic polymorphism of ABCB1 may influence the in vivo processes of lenalidomide.

Plus, there is significant interpatient variability in the plasma concentration of lenalidomide. Studies have found that the pharmacokinetic (PK) parameters of lenalidomide do not affect its efficacy but may be correlated with its toxicity^20,21^. Therefore, population pharmacokinetic (PPK) studies on lenalidomide are needed to identify factors contributing to interpatient PK differences, thus refining regimens to mitigate its adverse reactions. Yet hardly any of the current PPK studies has included as a covariate the genetic polymorphism of the ABCB1 gene^22–25^, we utilize a nonlinear mixed-effects model (NONMEM) to conduct a PPK study on lenalidomide in Chinese patients incorporating the genotypes of ABCB1 3435 C > T (rs1045642), ABCB1 1236 A > G (rs1128503) and ABCB1 2677 A > C/T (rs2032582) which have high variant frequencies in the Chinese population in the hope of optimizing the clinical application of lenalidomide^26^.

## Methods

### Patient eligibility

This was a prospective, open-label PPK study on lenalidomide in patients with different diagnosis. Patients were eligible if they were above the age of 18 and received lenalidomide treatment between October 2021 and June 2023 in the First Affiliated Hospital of Shandong First Medical University. Patients were excluded if (i) they had been included in other clinical trials, (ii) their clinical data were deemed insufficient, or (iii) there appeared other factors that rendered them ineligible.

This PPK study complied with the Helsinki Declaration and other relevant legal regulations, which was registered with Clinical Trials.gov (ID: NCT06069024 ) and approved by the Ethics Committee of the First Affiliated Hospital of Shandong First Medical University (code number: YXLL-KY-2021 (048)). It should be noted that the study did not interfere with the patients’ treatment plans. Except for the discomfort during blood sampling, patients were not exposed to any risks. Prior to blood sampling, the potential benefits and risks were thoroughly explained to the patients and their informed consent was obtained.

### Sample collection

Lenalidomide was administered at a dose of 10 mg or 25 mg per day. After five consecutive doses, blood samples were collected using opportunistic sampling and centrifuged at 6000 rpm for 10 min. The upper plasma layer was extracted for determination of lenalidomide concentrations while the lower blood cells were used for genotype analysis.

### Measurements

The chromatographic separation was performed on a LC-20ADXR series HPLC chromatograph (Shimadzu, Japan) with a Inertsil ODS-4 5µm, 2.1 × 100 mm chromatographic column (GL Sciences, Shanghai), with column temperature 40℃ and flow rate 0.45 mL/min. The mobile phase contained 1 mL formic acid in 1000 mL water (A) and acetonitrile (B). The linear gradient had the following program: 0.01 min (97:3) → 2 min (60:40) → 2.2 min (40:60) → 3.2 min (10:90) → 10.6 min (97:3). A Triple QuadTM 4500MD mass spectrometer (AB Sciex Company, the USA) was used for the detection and quantifcation of lenalidomide. The ion source used a positive ion electrospray ionization (ESI) source in a multi-reaction monitoring mode. The ion pair of lenalidomide was m/z 260.2 → m/z 149.0. The various condition parameters of the MS system were as follows: the ion spray voltage (IS) was 5500V, air curtain gas (CUR) pressure was 40 psi, ion source temperature (TEM) was 550℃, the collision energy (CE) was 20 V, the collision exit voltage (CXP) was 7V, the declustering potential (DP) was 92.12V, the atomization gas pressure (GS1) was 50 psi, the auxiliary gas pressure (GS2) was 50 psi.

Lenalidomide plasma concentrations were determined using high-performance liquid phase tandem mass spectrometry (HPLC–MS/MS). The standard curve equation was Y = (7.17223 × 10^–4^) X + 4.15187 × 10^–4^ (R = 0.99838), with a linear range between 2–1000 ng/mL and a lower limit of quantification of 2 ng/mL. The accuracy of the low, medium and high concentration quality control samples ranged from 95.68% to 108.22%, 90.06% to 97.00% and 91.23% to 97.28%, respectively. The intra-day and inter-day precision were both less than 15%.

Genotype was determined by using first-generation DNA sequencing technology. DNA from the patients was extracted using a DNA extraction kit (TIANGEN BIOTECH, Beijing). Firstly, primers were designed for ABCB1 3435 C > T, 1236 A > G and 2677 A > C/T. Then, the polymerase chain reaction (PCR) product was amplified and purified, and the purified PCR products were re-amplified subsequently. Finally, a ABI3730XL sequencer (Applied Biosystems, the USA) was used to sequence the amplification products. The representativeness of the included gene loci was assessed using Hardy–Weinberg equilibrium.

### PPK model

The PPK characteristics and simulations of lenalidomide were analyzed in a nonlinear mixed-effects model program (NONMEM V7.4, Icon Development Solutions, Ellicott City, MD, USA). NONMEM runs were executed by using Wings for NONMEM and R software (V4.2.0). The model development consisted of three steps: (i) establishment of the structural model; (ii) covariate analysis; (iii) evaluation of the final model.

The one-compartment and two-compartment models were tested and compared based on objective function value (OFV) and goodness-of-fit diagnostic plots to determine the optimal structural model. Between-subject variability (BSV) was described using a power exponential error model, expressed as: θ_i_ = θ_pop_ × Exp(η_i_). Here, θ_i_ represented the PK parameter value for the i-th subject, θ_pop_ denoted the population typical value of the pharmacokinetic parameter, and η_i_ signified BSV, following a normal distribution with a mean of 0 and a variance of ω2. Four residual error models (additive, proportional, power exponential and combined) were applied to both the one-compartment and two-compartment models to identify the most suitable model for describing residual variability.

Forward inclusion and backward elimination methods were used to investigate the influence of the following variables on PK parameters: age, sex, height, body weight, body surface area (BSA), aspartate aminotransferase (AST), alanine aminotransferase (ALT), total bilirubin (TBIL), total protein (TP), albumin (ALB), globulin (GLB), albumin/globulin ratio (A/G), serum creatinine (Scr), creatinine clearance (Ccr), cystatin C (CYS-C), blood urea nitrogen (BUN), uric acid (UA), hemoglobin (HG), β2 microglobulin (β2-MG), serum calcium concentration (CA), disease type, diet(taking lenalidomide on fasting or fed states), treatment regimen, concomitant medications and gene polymorphisms. In the forward inclusion process, if the inclusion of a covariate resulted in a decrease in OFV greater than 3.84, it was considered statistically significant (P < 0.05). The backward elimination process used a more stringent P-value criterion (P < 0.01), meaning that if the removal of a covariate led to an increase in OFV greater than 6.6, it was considered statistically significant.

The goodness-of-fit was assessed using diagnostic scatter plots, including observed concentrations/development values(DV) versus population predicted concentrations (PRED), DV versus individual predicted concentrations (IPRED), conditional weighted residuals (CWRES) versus TIME and CWRES versus PRED plots. The selection criteria for diagnostic plots were the same as those used in the structural model construction.

The stability of the final model was evaluated using a bootstrap method. One thousand new datasets were generated by resampling the original data with replacement and corresponding model parameters were estimated for each dataset. The median and 5th and 95th percentiles of the bootstrap parameters were calculated.

The predictability of the final model was assessed by using the normalized prediction distribution errors (NPDE) test. One thousand simulations were performed based on the final model parameters using the dataset. The NPDE values were expected to follow a (0,1) normal distribution. The results of NPDE were summarized graphically using R software, including quantile–quantile (Q-Q) plots of NPDE, NPDE histograms, NPDE versus TIME plots and NPDE versus PRED plots.

### Safety analysis

All adverse events (AEs) occurring were collected during the lenalidomide treatment cycle where the blood samples were taken, and the severity of adverse reactions was assessed using the Common Terminology Criteria for Adverse Events version 5.0 (CTCAE v5.0) published by the U.S. Department of Health and Human Services in 2017. AEs higher than grade 3 were defined as serious AEs and the rest as non-serious AEs.

### Statistical analysis

The safety analysis was based on covariant results from the PPK model and patients whose safety measurements were available. IBM SPSS Statistics 26.0 (IBM Corporation, Armonk city, New York State,USA) was used for performing the statistical analyses. Differences in the continuous variables between groups were evaluated by using Mann–Whitney U test, and differences in the categorical variables between groups were evaluated by using Fisher’s exact test.

### Ethical approval

All procedures performed in studies involving human participants were in accordance with the ethical standards of the institutional and/or national research committee and with the 1964 Declaration of Helsinki and its later amendments or comparable ethical standards. This study was approved by the ethics committee of the First Affiliated Hospital of Shandong First Medical University (ethics approval number: YXLL-KY-2021 (048)).

### Consent to participate

Written informed consent for this study was obtained from all the patients.

### Consent to publish

Written informed consent for publication of this study was obtained both from the patients and their family.

## Results

### Patients.

A total of 51 patients were included in this study, with a collection of 87 blood samples. All patients were of Han ethnicity, including 33 patients diagnosed with MM, 17 patients diagnosed with Lymphoma and 1 patient diagnosed with MDS, among whom 27 patients received bortezomib-lenalidomide-dexamethasone (VRD) regimen, 12 patients received rituximab-lenalidomide (RR) chemotherapy and 12 patients received other regimens containing lenalidomide. The genotype detection rate for all three gene loci in the patients was 100% and the genotype distribution complied with the Hardy–Weinberg equilibrium. Detailed information on continuous variables of the patients is shown in Table 1, information on categorical variables is presented in Table 2, and genetic data are provided in Table 3.

**Table 1:** Information on continuous variables of the patients (n = 51). A/G albumin/globulin ratio.

Categories	Mean ± SD	Median (range)
Age (year)	65.2 ± 10.8	67.0 (34.0–85.0)
Height (cm)	166 ± 8.48	167 (150–181)
Weight (kg)	67.5 ± 11.0	70.0 (40.0–90.0)
BSA (m^2^)	1.79 ± 0.180	1.79 (1.35–2.17)
AST (U/L)	23.6 ± 19.9	17.7 (4.80–98.5)
ALT (U/L)	27.8 ± 28.8	17.9 (4.50–155)
TBIL (µmol/L)	11.1 ± 5.62	9.90 (4.60–31.2)
TP (g/L)	62.9 ± 11.2	60.1 (43.7–97.6)
ALB (g/L)	35.9 ± 5.32	36.9 (23.4–47.8)
GLB (g/L)	27.3 ± 12.1	23.3 (13.7–71.8)
A/G	1.55 ± 0.660	1.49 (0.360–2.99)
Scr (µmol/L)	67.9 ± 24.2	63.0 (30.0–140)
Ccr (ml/min)	91.1 ± 33.2	87.4 (33.3–191)
CYS-C (mg/L)	1.17 ± 0.449	1.07 (0.470–3.09)
BUN (mmol/L)	5.44 ± 2.14	5.00 (2.50–13.6)
UA (µmol/L)	288 ± 93.4	288 (99.0–550)
HG (g/L)	105 ± 19.6	106 (57.0–153)
β2-MG (mg/L)	2.14 ± 0.183	2.96 (1.07–10.8)
CA (mmol/L)	3.33 ± 1.81	2.16 (1.51–2.52)

**Table 2:** Information on categorical variables of the patients (n = 51).

Categories	Numbers	Proportions (%)
Sex		
Men	27	52.9
Women	24	47.1
Disease types		
MM	33	64.7
Lymphoma	17	33.3
MDS	1	2.00
Treatment regimens		
VRD	27	52.9
RR	12	23.5
Others	12	23.5
Other concomitant medications		
Aspirin	28	54.9
Omeprazole	27	52.9
Acyclovir	23	45.1
Glutathione	16	31.4
Tiopronin	14	27.4
Mecobalamin	11	21.6
P-gp substrates	7	13.7
Other renally excreted drugs	38	74.5
Diet		
Postprandial medication	28	54.9
Fasted medication	23	45.1

**Table 3:** Genotypes and genetic equilibrium test results for each gene locus (n = 51).

Gene loci	Genotypes	Frequency (%)	Alleles	Frequency (%)	*P* value
ABCB1 3435 C > T	CC	19 (37.2%)	C	59 (58.9%)	0.27
	CT	21 (41.2%)	T	43 (41.1%)	
	TT	11 (21.6%)			
ABCB1 1236 A > G	AA	24 (47.1%)	A	66 (68.6%)	0.99
	AG	22 (43.1%)	G	24 (31.4%)	
	GG	5 (9.80%)			
ABCB1 2677 A > C/T	AA	12 (23.5%)	A	44 (43.1%)	0.15
	AC/AT	20 (39.2%)	C	46 (45.1%)	
	CC/CT/TT	19 (37.3%)	T	12 (11.8%)	

### PPK model establishment

After comprehensive comparison of OFV and diagnostic plots for goodness of fit, a one-compartment model with first-order absorption and elimination described by an additive residual model was selected as the structural model. The PPK parameters included the apparent clearance (CL/F), volume of distribution (V/F) and absorption rate constant Ka. Due to the limit of plasma concentration data collected in this study, the value of Ka could not be robustly estimated, and thus Ka was fixed at 6.55 h^−1^ based on existing lenalidomide PPK studies^23–25^.

Covariates were sequentially included using the forward inclusion and backward elimination methods. The final retained covariates were ABCB1 3435 C > T genotype and diet, and both of them had significant impact on V/F, while none covariate for the CL/F could be found. The final formula was: V/F_i_ (L) = 29.1 × Exp(0.554 + θ_ABA_ × COVR_ABAi_ + θ_CHFY_ × COVR_CHFYi_) ( θ_ABA_ was the coefficient corresponding to each genotype of ABCB1 3435 C > T, COVR_ABAi_ was the individual covariate value for the i-th individual; CHFY representsed whether or not taking lenalidomide within 1 h after a meal, and θ_CHFY_ was the coefficient corresponding to diet, COVR_CHFYi_ was the individual covariate value for the i-th individual).

**Figure 1:**
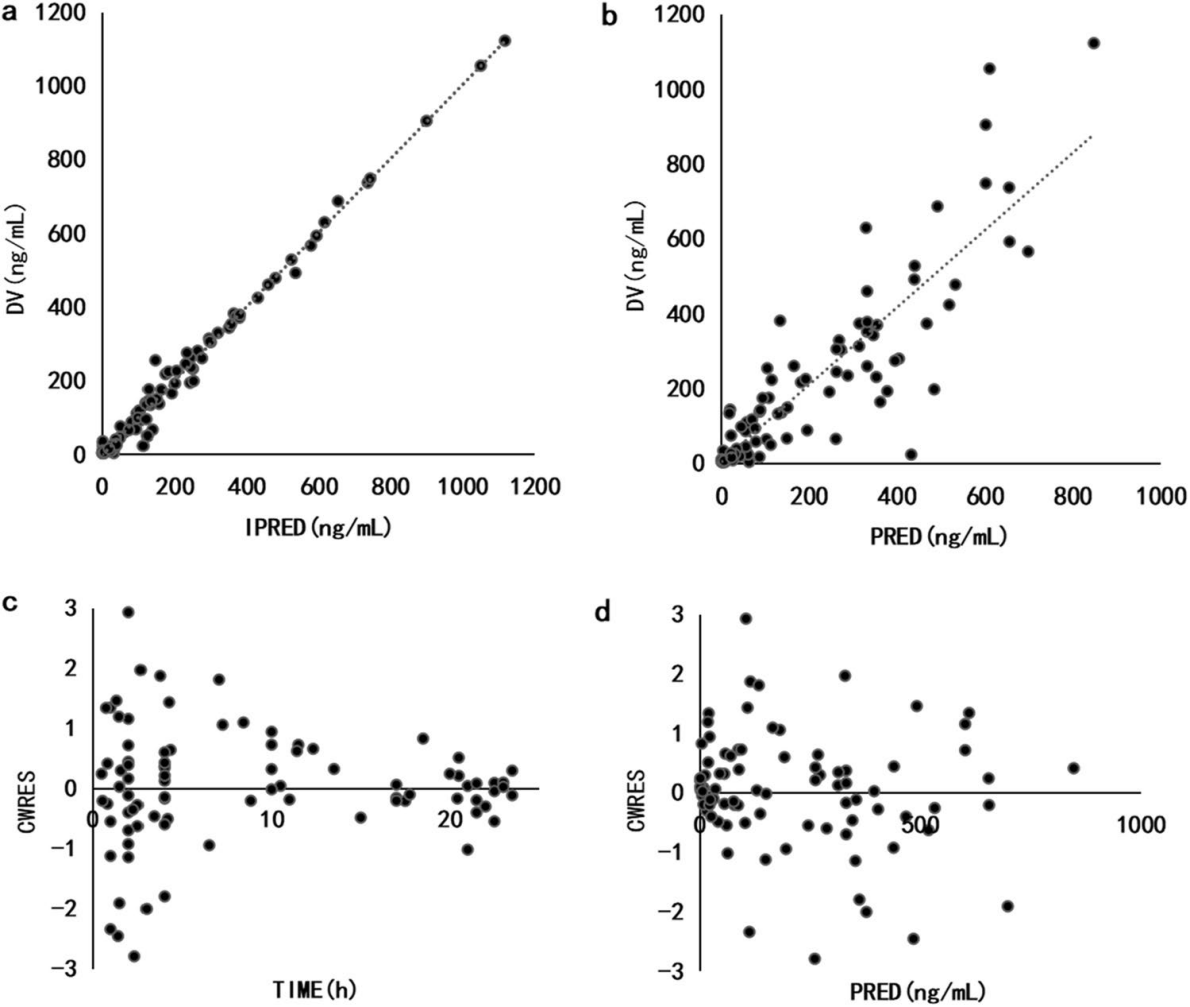
Goodness-of-fit plots for the final model. (**a**) DV versus IPRED; (**b**) DV versus IPRED; (**c**) CWRES versus TIME; (**d**) CWRES versus PRED. The dotted line represents the line y = x.

The model diagnostic plots of the final model showed that most data points were evenly distributed on both sides of the y = x line in the DV versus IPRED plot and DV versus PRED plot, indicating that the IPRED and PRED could well reflect the observed concentrations. The CWRES versus TIME plot and the CWRES versus PRED plot the final model showed symmetrical distribution of data points between the y =  ± 2 lines, with y = 0 as the axis of symmetry, which suggested that the chosen residual model was appropriate. Overall, these results indicated a good fit of the final model (Fig. 1).

According to the results of 1000 bootstrap iterations of the final model, the robustness of the model reached 78.7%. The estimated values of each parameter in the final model were within the 5% to 95% confidence interval of the bootstrap parameters and were close to the median values of the bootstrap parameters, indicating positive stability of the final model. The parameter estimates of the final model and the bootstrap results were presented in Table 4.

**Table 4:** Results of the parameter estimates of the final model and the bootstrap parameter values. θ denoted the coefficient corresponding to each parameter. η denoted BSV; ε denoted intra-subject variation of the additive type. RSE denoted relative standard error.

Parameters	Final model		Bootstrap	
	Estimates	RSE (%)	Median	5%-95% CI
θ_Ka_(h^−1^)	6.55 (fixed)		6.55 (fixed)	
θ_CL/F_(L/h)	7.25	7.10	7.25	(6.23–8.56)
θ_V/F_(L)	29.1	9.90	28.4	(17.5–57.2)
θ_ABA_				
ABCBA 3435 C > T = CT	0.0151	42.2	0.0151	(0.0151–0.140)
ABCBA 3435 C > T = TT	0.335	63.9	0.350	(0.0151–1.50)
θ_CHFY_	0.558	24.4	0.569	(-0.980–1.18)
η_CL/F_(%)	35.5	17.1	31.0	(0.300–43.5)
η_V/F_(%)	55.4	17.0	53.4	(28.3–96.8)
ε(ng/mL)	38.5	21.6	39.9	(20.8–62.9)

**Figure 2:**
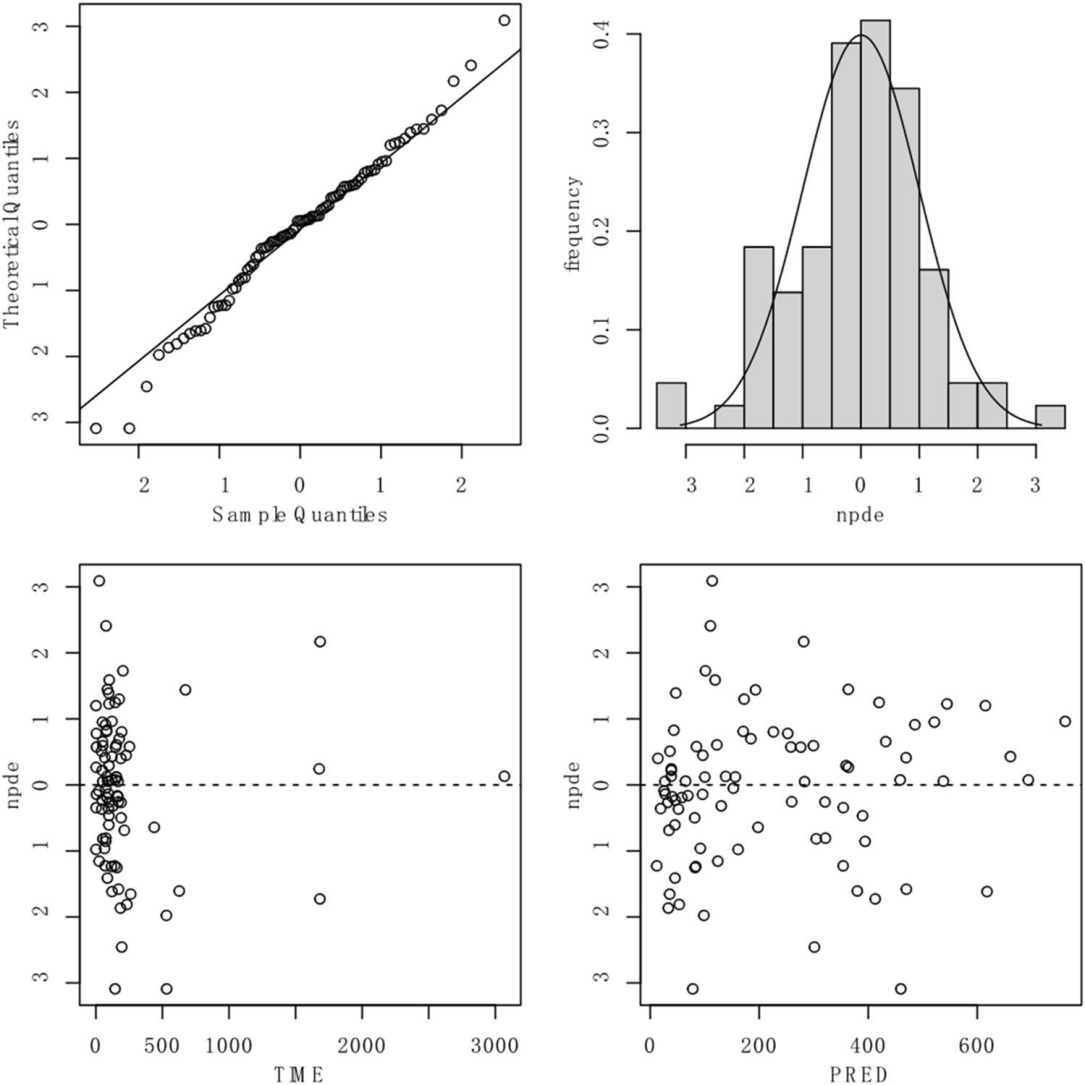
The graphical results of the NPDE test for the final model. Upper left the Q-Q plot. Upper right the NPDE histogram. Bottom left the scatter plot of NPDE versus TIME. Bottom right the scatter plot of NPDE versus PRED.

The mean and variance of NPDE were -0.0692 and 1.3 respectively, with Wilcoxon signed rank test *P* = 0.721, Shapiro-Wilks test of normality *P* = 0.569, and Fisher variance test *P* = 0.0638, which indicated that the NPDE followed a normal distribution. The graphical summary of the NPDE results was shown in Fig. 2. The Q-Q plot displayed data points tightly clustered around the trend line and evenly distributed on both sides. The NPDE histogram approximated a normal distribution, and the scatter plots of NPDE versus TIME and NPDE versus PRED showed no significant trend changes in the data points. Considering the statistical and graphical results collectively, it could be concluded that the final model possessed a certain level of predictability.

### Safety study

Among the 51 patients, one died due to serious underlying disease and safety data were lacking for 11 patients, so safety analysis was finally conducted on the remaining 39 patients. The most common AEs observed were lymphocyte percentage (LY%) decrease (41.0%), neutrophil percentage (NE%) increase (35.9%), lymphocyte count (LY#) decrease (35.9%), neutrophil count (NE#) increase (25.6%), platelet decrease (23.1%), hypocalcemia (23.1%), hypokalemia (20.5%) and anemia (15.4%). Nine patients experienced grade 3–4 AEs, with the most frequent being lymphocyte count decrease (13.2%). Among them, 5 patients had at least two different grade 3–4 AEs. For detailed safety information, see Table 5.

**Table 5:** AEs in patients undergoing lenalidomide therapy (n = 39).

AEs	Grade 1–4	Grade 3–4
Hematological system		
LY% decrease	16 (41.0%)	0
NE% increase	14 (35.9%)	0
LY# decrease	14 (35.9%)	5(12.8%)
NE# increase	10 (25.6%)	0
Platelet decrease	9 (23.1%)	2(5.13%)
Anemia	6 (15.4%)	1(2.56%)
Leukocyte decrease	5 (12.8%)	0
NE# decrease	3 (7.69%)	0
Red blood cells decrease	3 (7.69%)	0
NE% decrease	2 (5.13%)	0
Non-hematological system		
Hypocalcemia	9 (23.1%)	0
Hypokalemia	8 (20.5%)	1 (2.56%)
Fever	5 (12.8%)	0
Diarrhea	4 (10.3%)	1 (2.56%)
Hypomagnesemia	4 (10.3%)	1 (2.56%)
Hypoalbuminemia	3 (7.69%)	0
Edema of lower extremity	3 (7.69%)	0
Hyponatremia	2 (5.13%)	0
Cough	2 (5.13%)	0
Fungal infection of lung	2 (5.13%)	2 (5.13%)
Fatigue	2 (5.13%)	1 (2.56%)
Pruritus	2 (5.13%)	0
Hand numbness	1 (2.56%)	0
Deep vein thrombosis	1 (2.56%)	1 (2.56%)
Drowsiness	1 (2.56%)	0
Nausea	1 (2.56%)	0

NONMEM software was used to predict V/F in 39 patients, with V/F ranging from 15.98–236.8 L. Patients were divided into serious and non-serious AEs groups according to the severity of AEs, and the correlation in V/F between the two groups was compared by using the Mann–Whitney U test, which showed that the V/F value in serious AEs group was significantly higher than that in non-serious AEs group (median = 67.04 L vs 37.17 L, *P* = 0.033).

Further investigation on the correlation between covariate for V/F and serious AEs was carried out. The result indicated that there was no difference in the incidence of lenalidomide-related serious AEs between patients in the fasting medicine and postprandial medicine groups (*P* = 0.251), while patients carrying the genotype CT or TT at ABCB1 3435 C > T locus had a higher incidence of serious AEs compared to those with genotype CC (*P* = 0.039). In addition, according to the covariant equation of the final model, the V/F, from the smallest to the largest, was as follows: (i) V/F was 50.64 L when patients carried genotype CC at ABCB1 3435 C > T locus with fasted medication; (ii) V/F was 51.41 L when patients carried genotype CT with fasted medication; (iii) V/F was 88.48 L when patients carried genotype CC with postprandial medication; (iv) V/F was 89.82 L when patients carried genotype CT with postprandial medication; (v) V/F was 98.96 L when patients carried genotype TT with fasted medication; (vi) V/F was 172.9 when patients carried genotype TT with postprandial medication. It was found that patients carrying genotype TT at the ABCB1 3435 C > T locus and patients carrying genotype CT with postprandial medication were more likely to suffer from serious AEs than other patients (*P* = 0.019). What is more, patients who carried genotype TT with taking lenalidomide within 1h after a meal had the highest V/F, and they also had a more possibility to suffer from serious AEs (*P* = 0.037). See Table 6.

**Table 6:** Comparison of the number of patients in different lenalidomide AEs groups (n = 39). *Between-group differences based on Fisher’s exact test.

Groups	No Grade 3–4 AE (n)	Grade 3–4 AE (n)	P Value*
ABCB1 3435 C > T			
CC	13	0	
CT	13	5	
TT	4	4	
CC:CT			0.058
CC:TT			0.012
CT:TT			0.382
CC: (CT + TT)			0.039
CT:(CC + TT)			0.706
TT:(CC + CT)			0.065
Diet			
Fasted medication	15	2	0.251
Postprandial medication	15	7	
ABCB1 3435 C > T + diet			
(i) CC + Fasted medication	6	0	
(ii) CT + Fasted medication	8	2	
(iii) CC + Postprandial medication	7	0	
(iv) CT + Postprandial medication	5	3	
(v) TT + Fasted medication	1	0	
(vi) TT + Postprandial medication	3	4	
(i): [(ii) + (iii) + (iv) + (v) + (vi)]			0.305
[(i) + (ii)]: [(iii) + (iv) + (v) + (vi)]			0.262
[(i) + (ii) + (iii)] : [(iv) + (v) + (vi)]			0.019
[(i) + (ii) + (iii) + (iv)] : [(v) + (vi)]			0.065
[(i) + (ii) + (iii) + (iv) + (v)] : (vi)			0.037

## Discussion

PPK studies on lenalidomide have been quite limited so far. In 2013, a study by Chen et al.^22^demonstrated that Ccr was the only covariate for CL/F of lenalidomide, and factors including age, body weight, gender, race (Caucasian, Asian and others) and mild hepatic impairment had no influence on its PPK. A study focusing on patients with MM identified Ccr and BSA as covariates for lenalidomide’s CL/F and V/F, respectively^23^, which recommended that patients with BSA less than 1.8 m^2^ use a lower dose of lenalidomide than the standard 25 mg/day. Connarn et al.^24^ developed a PPK model of lenalidomide in patients and healthy volunteers, where the former had lower CL/F and V/F than the latter, and identified Ccr and body weight as two covariates. Additionally, Hughes et al.^25^ also emphasized the significant impact for Ccr on lenalidomide’s CL/F.

Different from previous studies, this study included particular variables including disease type, diet, concomitant medications and genetic polymorphisms in addition to demographic characteristics, laboratory test results and so on. Attempts were made to fix the value of Ka based on existing lenalidomide PPK studies at 6.55 h^−1^, which resulted in a well-fitting, stable and predictive model^23–25^. The final model failed to find a covariate for the CL/F of lenalidomide, and just revealed a correlation between lenalidomide's V/F and the ABCB1 3435 C > T genotype as well as diet, which was different from the previous studies^23–25^, implying that genotype of ABCB1 3435 C > T and diet may had a greater impact on the PK process of lenalidomide than any other factors.

In addition, we also explored the correlation between PK parameters and the occurrence of serious AEs, and we found that patients with a higher V/F of lenalidomide were more likely to experience 3–4 grade AEs, speculating a higher V/F value meant a longer drug retention time and a longer toxic reactions. Kobayashi et al.^27^ found that patients carrying the CT or TT genotype at the ABCB1 3435 C > T locus had significantly higher area under the concentration–time curve from 0 to 24 h (AUC_0-24_) compared to patients with the CC genotype. Additionally, based on univariate and multivariate regression models, it was predicted that patients carrying the CT or TT genotype at this locus were more likely to experience serious adverse reactions. Similarly, we found that the genotype at the ABCB1 3435 C > T locus, as well as diet, was also associated with the incidence of lenalidomide-related serious AEs, but the PK parameter it influenced was V/F rather than AUC_0-24_, the mechanism behind which needs more researches to reveal further. As the results indicated, V/F was highest in patients who carried genotype TT at the ABCB1 3435 C > T locus with taking lenalidomide within 1 h after a meal, and such patients may be more prone to suffer from serious AEs than others. Although the factors we found affecting lenalidomide serious AEs were not completely consistent with those found by Kobayashi, the association of ABCB1 3435 C > T with serious AEs is indisputable and warrants further study in the future. Ingrid et al.^28^ found that patients with ABCB1 1199 G > A in the presence of the A base variant had a longer time to disease progression. Furthermore, patients with the ABCB1 2677 TT and ABCB1 3435 TT genotypes were more likely to have outcomes superior to very good partial remission when first treated with lenalidomide, most of whom were younger and more likely to have received a prior stem cell transplant. However, no significant associations were found between genotype and hematological AEs. Due to the inconsistency in the type of disease, disease duration and treatment regimen of the patients, we were unable to compare genotype with lenalidomide efficacy, which was one of the shortcomings of our study.

## Conclusion

The first PPK model of lenalidomide specifically for the Chinese population was established in this study, which indicated that the covariates influencing the V/F of lenalidomide were ABCB1 3435 C > T gene polymorphism and diet. We speculate that patients carrying genotype CC at ABCB1 3435 C > T locus are more likely to be safe to take lenalidomide. However, patients with ABCB1 3435 C > T genotype variant being TT and taking lenalidomide within 1 h after a meal may be prone to serious AEs and need to be further monitored while taking medicine. However, given a relatively small sample size, the present results should be interpreted with caution and investigated further in a larger study population.

## Data Availability Statement

The datasets generated and/or analyzed during the current study are available in the OSF repository (https://osf.io/9m2hz), and DNA-seq fastq files were deposited in NCBI Sequence Read Archive (SRA) under accession number PRJNA1053270.

## Abbreviations

PK: Pharmacokinetics
PPK: Population pharmacokinetics
AEs: Adverse events
MDS: Myelodysplastic syndromes
MM: Multiple myeloma
P-gp: Glycoprotein
ABCB1: ATP-binding cassette subfamily B member 1
NONMEM: Nonlinear mixed-effects model
OFV: Objective function value
BSV: Between-subject variability
BSA: Body surface area
AST: Aspartate aminotransferase
ALT: Alanine aminotransferase
TBIL: Total bilirubin
TP: Total protein
ALB: Albumin
GLB: Globulin
A/G: Albumin/globulin ratio
Scr: Serum creatinine
Ccr: Creatinine clearance
CYS-C: Cystatin C
BUN: Blood urea nitrogen
UA: Uric acid
HG: Hemoglobin
β2-MG: β2 Microglobulin
CA: Serum calcium concentration
PCR: Polymerase chain reaction
DV: Observed concentrations/development values
PRED: Population predicted concentrations
IPRED: Individual predicted concentrations
CWRES: Conditional weighted residuals
NPDE: Normalized prediction distribution errors
